# Extensive validation of CM SAF surface radiation products over Europe

**DOI:** 10.1016/j.rse.2017.07.013

**Published:** 2017-09-15

**Authors:** Ruben Urraca, Ana M. Gracia-Amillo, Elena Koubli, Thomas Huld, Jörg Trentmann, Aku Riihelä, Anders V. Lindfors, Diane Palmer, Ralph Gottschalg, Fernando Antonanzas-Torres

**Affiliations:** a EDMANS Group, Department of Mechanical Engineering, University of La Rioja, Logroño, Spain; b European Commission, Joint Research Centre, Via E. Fermi 2749, Ispra 21027, Italy; c Centre for Renewable Energy Systems Technology, Loughborough University, LE11 3TU Leicestershire, UK; d Deutscher Wetterdienst, Offenbach 63067, Germany; e Finnish Meteorological Institute, P.O. Box 503, Helsinki FI-00101, Finland

**Keywords:** Satellite-based models, Global horizontal irradiance, CM SAF, Solar radiation data, Pyranometer

## Abstract

This work presents a validation of three satellite-based radiation products over an extensive network of 313 pyranometers across Europe, from 2005 to 2015. The products used have been developed by the Satellite Application Facility on Climate Monitoring (CM SAF) and are one geostationary climate dataset (SARAH-JRC), one polar-orbiting climate dataset (CLARA-A2) and one geostationary operational product. Further, the ERA-Interim reanalysis is also included in the comparison. The main objective is to determine the quality level of the daily means of CM SAF datasets, identifying their limitations, as well as analyzing the different factors that can interfere in the adequate validation of the products.

The quality of the pyranometer was the most critical source of uncertainty identified. In this respect, the use of records from Second Class pyranometers and silicon-based photodiodes increased the absolute error and the bias, as well as the dispersion of both metrics, preventing an adequate validation of the daily means. The best spatial estimates for the three datasets were obtained in Central Europe with a Mean Absolute Deviation (MAD) within 8–13 W/m^2^, whereas the MAD always increased at high-latitudes, snow-covered surfaces, high mountain ranges and coastal areas. Overall, the SARAH-JRC's accuracy was demonstrated over a dense network of stations making it the most consistent dataset for climate monitoring applications. The operational dataset was comparable to SARAH-JRC in Central Europe, but lacked of the temporal stability of climate datasets, while CLARA-A2 did not achieve the same level of accuracy despite predictions obtained showed high uniformity with a small negative bias. The ERA-Interim reanalysis shows the by-far largest deviations from the surface reference measurements.

## Highlights

• Validation of daily means of CM-SAF satellite products over 313 ground stations
• SARAH provides the best estimations of solar radiation over Europe.
• Low quality pyranometers are a significant uncertainty source in the validation.

## 1 Introduction

High-quality solar radiation data is demanded in several fields, such as climate analysis, hydrology, agriculture or solar energy production. The most accurate method to obtain irradiance values at surface level is with ground radiometers. However, the quality of these records depends on the type of radiometer (ISO and WMO classifications (ISO, 1990, WMO, 2008)), the calibration process and the regular maintenance of the equipment (Vuilleumier et al., 2014). Moreover, ground stations are sparse, specially the ones with high-quality and well-maintained equipment, and the temporal coverage varies among stations. Hence, different methods have been developed to estimate the incoming solar radiation from the historical empirical correlations with meteorological variables to more advanced techniques such as interpolation, reanalysis and satellite-based models (Bojanowski et al., 2014, Urraca et al., 2016).

Satellite-based methods have reached a high degree of maturity and are becoming the most common option to evaluate solar radiation (Polo et al., 2016). They provide consistent estimations since the 1980s, with global coverage and resolutions up to 15 min and a few kilometers. Models can be based on images from either *geostationary satellites* (e.g. Meteosat, GOES and GMS), or *polar-orbiting satellites* (e.g. NOAA series and Metop). Geostationary-based products have higher temporal resolution (up to 15 min) but limited spatial coverage (± 65° latitude). In contrast, polar-orbiting products have global coverage but lower temporal resolution, limited to daily means at lower latitudes. Regardless of the type of image used, three main approaches exist to derive surface irradiance from the satellite image (Sengupta et al., 2015). *Empirical models* use experimental correlations between the pixels of the satellite image and the atmospheric transmissivity. *Physical models* solve radiative-transfer equations at the different layers of the atmosphere. Hence, they are computationally more expensive and require a precise knowledge of the composition of the atmosphere. *Semi-empirical models* have emerged as an hybrid approach between the prior two methods.

While historically most methods were purely empirical (i.e. Heliosat based models), newer models have been increasingly including some type of radiative transfer model (RTM) computations (Qu et al., 2016). The cloud-index is empirically derived from the visible channels of the satellite and then used to modify a clear-sky RTM. This shift to a more physical approach was enabled by the higher availability of ancillary products that describe the state of the atmosphere (mainly aerosols and water vapor), as well as advances on the computational field. One of the most common computational solutions is to save RTM clear-sky computations in look-up tables (LUTs) and then use parameterizations to obtain the actual clear-sky values (Mueller et al., 2012, Qu et al., 2016). Currently, most of the available options follow this semi-empirical approach and use geostationary satellite images as the main input.

Finally, regarding the implementation of the model, two types of datasets can be found. *Climatological datasets* are obtained by processing long periods of satellite images with the same model and a unique set of inputs. They are useful for climate applications as they guarantee the consistency of the dataset obtained. In contrast, *operational products* provide real-time estimates and may undergo upgrades in some of the inputs or in the model itself. Different datasets or webpages services are available such as the CM SAF products (CM SAF, 2015), also available via PVGIS webpage over Europe and Asia (PVGIS, 2016); LSA SAF products (LSA SAF, 2015); HelioClim-3 from MINES ParisTech and available via the SODA Service (SoDa, 2016); HelioMont from MeteoSwiss (Castelli et al., 2014); the SOLEMI and DLR-ISIS datasets from the DLR (DLR, 2016); the MACC-RAD product based on the new Heliosat-4 from the MACC project and Copernicus program (Qu et al., 2016); the National Solar Radiation Database (NSRDB) from NREL (NREL, 2016), the NASA/GEWEX Surface Radiation Budget (SRB) (NASA/GEWEX, 2016) or the SolarGIS database from GeoModel Solar (GeoModel Solar, 2017). The reader is referred to some recent reviews for a more detailed analysis of the available resources (Vernay et al., 2014, Ineichen, 2014, Sengupta et al., 2015, Polo et al., 2016)

Due to these advances in satellite-based methods, many application and validation studies have been published during the last years (Gracia Amillo et al., 2014, Antonanzas-Torres et al., 2013, Sanchez-Lorenzo et al., 2013, Zak et al., 2015, Gracia Amillo et al., 2015, Riihelä et al., 2015). However, in most of these works the assessment against ground records is performed in a reduced set of stations, mainly the BSRN or local networks. Hence, our main goal is to provide an extensive analysis of different types of satellite-based datasets using a high density of ground stations over Europe. We have selected three independent datasets from the Satellite Application Facility on Climate Monitoring (CM SAF) (Schulz et al., 2009). The first two, the geostationary satellite-based SARAH (Muller et al., 2015) and the polar-orbiting satellite-based CLARA-A2 (Karlsson et al., 2016), are climate records. The third dataset is the CM SAF operational product based on the SEVIRI instruments onboard the geostationary Meteosat satellites. In addition, the ERA-Interim reanalysis is included in this evaluation (Dee et al., 2011, Boilley and Wald, 2015). All products are evaluated with a ground dataset composed by 313 stations over Europe, from 2005 to 2015. The variable validated is the global horizontal irradiance (GHI), as it is the value typically available at on-ground stations, and the study focuses on the following aspects: impact of geographical location (latitude, elevation, continentality), inter-annual and intra-annual variability, influence of the type of radiometer and quantification of some uncertainties within the validation process.

The organization of the paper is as follows. In Section 2 the radiation datasets used in the study are presented, while in Section 3 the ground records used as reference data are described. In Section 4 quality control, data aggregation, data merging and validation procedures are explained. In Section 5 the results obtained are shown and discussed. The main aspects analyzed are the uncertainty of estimates, the spatial distribution of errors, the inter-annual and intra-annual variability, and the influence of the type of sensor. Finally, in Section 6 the main conclusions and remarks are drawn.

## 2 Solar radiation products

### 2.1 SARAH-JRC

The SARAH solar radiation data record Müller et al. (2015) belongs to the class of *climate data records* of the CM SAF where the aim is to produce a long-term data set, which is homogeneous in time, that is, without changes in time due to changes in satellites or retrieval methods. SARAH has been derived using data from the MVIRI instruments onboard the Meteosat first generation satellites, MFG, (METEOSAT 2-7) and from the SEVIRI instruments onboard the Meteosat second generation, MSG, (Meteosat 8-10) satellites. Before 2006 the data are from MFG satellites while from 2006 onwards the MSG data have been used. The CM SAF SARAH data record provides the global and the direct surface solar radiation.

The retrieval of the surface solar radiation is performed using a modified Heliosat method to calculate the effective cloud albedo (CAL) and the SPECMAGIC clear-sky model described in Müller et al. (2012). The calculation of the clear-sky irradiance uses monthly average values of the total column of water vapor from ECMWF ERA-interim, and long-term monthly climatologies of aerosol optical depth based on MACC (Inness et al., 2013, Mueller et al., 2015).

SARAH has been validated by a number of authors (e.g., Müller et al., 2012, Gracia Amillo et al., 2014), using mainly high-quality ground stations such as those of BSRN.

The SARAH data used in the present study consists of solar irradiance values obtained from hourly satellite images, with no time averaging. This is the version used by the Joint Research Centre (JRC) for the photovoltaic energy calculator PVGIS (PVGIS, 2016). In this regard, these data differ from the version of SARAH currently available (December 2016) from CM SAF, where the hourly data have been obtained from a weighted average of half-hourly irradiance values. Also the method to derive daily mean surface solar radiation from the instantaneous satellite observations differs from the method used by the CM SAF.

### 2.2 CM SAF operational product

The CM SAF operational product of surface irradiance is generated on a daily basis with a temporal delay of about 10 days using the satellite information provided by the operational SEVIRI instrument onboard the Meteosat prime satellite. The retrieval algorithm is based on a look-up-table approach as described in Müller et al. (2009). Using the multi-spectral information from the SEVIRI instrument the cloud mask is determined; in the case of cloud-free conditions the surface irradiance is calculated using the MAGIC clear-sky surface irradiance algorithm (Müller et al., 2009). In the case of cloud coverage the measured reflected solar irradiance is related to the atmospheric optical depth based on a precalculated look-up table using additional boundary conditions, e.g., the surface albedo. Hourly data are used to derive daily means considering the diurnal cycle using the approach by Diekmann et al. (1998); the monthly means provided by CM SAF are calculated based on the daily means. The operational daily and monthly mean CM SAF products are available aggregated on a 15 km sinusoidal grid. In the present study, the instantaneous hourly data of surface irradiance are used. The CM SAF operational product of global radiation is validated regularly by the CM SAF using BSRN surface reference measurements.

### 2.3 CLARA-A2

The CM SAF CLARA-A2 data record is based on observations of the AVHRR instruments onboard the polar orbiting NOAA- and METOP-satellites. CLARA-A2 provides global information on cloud (coverage and properties), surface radiation (shortwave and longwave), and the surface albedo from 1982 to 2015 with a spatial resolution of 0.25° (Karlsson et al., 2016). The surface solar radiation data are derived with a look-up-table based on Müller et al. (2009) using auxiliary data of water vapor, surface albedo, and aerosol loading. Due to reduced data quality, no data is available over snow-covered surfaces. The surface irradiance has been validated against measurements from the BSRN surface reference network (Karlsson et al., 2016).

### 2.4 ERA-Interim reanalysis

ERA-Interim reanalysis (Dee et al., 2011) was released in 2011 by the European Center for Medium-range Weather Forecast (ECMWF) as the successor of the ERA-40. It has global coverage, no gaps, 3-hourly resolution and it can be freely accessed via the Meteorological Archival and Retrieval System (MARS). The data cover the period from 1979 to present (with a delay of one to two months), though it will be replaced during 2017 by a new version, the ERA-5. ERA-Interim surface radiation products are available in a regular longitude-latitude grid of 0.75° ×0.75°, which is a result of the interpolation from a reduced Gaussian grid N128 (approx. 79 km), where the actual computations are made.

## 3 Reference data: ground records

**Fig. 1:**
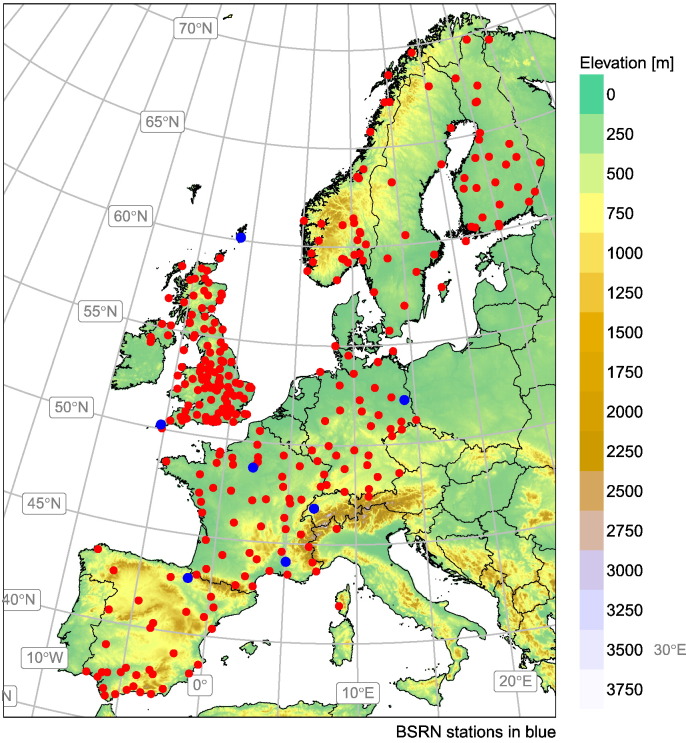
Locations of the 313 ground stations used in the present study. BSRN stations are plotted in blue.

The reference dataset, which covers the time period from 2005 to 2015 both inclusive, is composed of 313 ground stations located in several European countries spanning the full range of latitudes in Europe (Fig. 1). Data is retrieved at the highest temporal resolution available and all stations have at least one year of complete data (see Appendix 1). In most stations, only the GHI is available and consequently it is the only variable used in the validation and the quality control. The dataset is composed by records obtained with different type of pyranometers. Thermopile pyranometers, based on the thermoelectric effect, are the highest quality radiometers and are installed in most stations. Within thermopiles, the ISO-9060 (ISO, 1990) establishes three levels of quality: Secondary Standard (highest quality), First Class and Second Class. Besides, a few stations use silicon-based photodiodes, based on the photovoltaic effect. These pyranometers are non compliant of the ISO-9060 requirements due to the spectral response of the silicon, which is limited to 400–1100 nm.

Most stations belong to either meteorological or agricultural national networks (see Table 1). In Sweden, data from the Swedish Meteorological and Hydrological Institute (SMHI) (SMHI, 2016) is used. SMHI measurements are registered with a CM11 [Kipp&Zonen] pyranometer prior to 2008 and with a CM21 [Kipp&Zonen] afterwards. In both cases, the pyranometers are ventilated, which improves the performance of the sensor under extreme conditions with frost and snow. In Finland, data is provided by the Finnish Meteorological Institute (FMI) (FMI, 2016). FMI standard pyranometer is the CM11 [Kipp&Zonen] and all of them are ventilated as well. In Norway, records from the Landbruksmeteorologisk Tjeneste (LMT) are used. The LMT is a project run by the Norwegian Institute of Bioeconomy Research (NIBIO) for emergency services and agricultural research (NIBIO-LMT, 2016). Most of the selected stations are located along the shoreline, which is characterized by narrow fjords cutting into high mountains. The pyranometers installed in most stations are the CM11/CMP11 [Kipp&Zonen], but there is one station with the Second Class CM3 [Kipp&Zonen]. In UK, data from the national weather service (Met Office), accessible via the Met Office Integrated Data Archive System (MIDAS), are used. The network had some Second class pyranometers, the CM3 and CM5 [Kipp&Zonen], prior to 2010, but after that year all available records are from the CM11/CMP11 [Kipp&Zonen] pyranometer. In Germany, data from the meteorological office, Deutscher Wetterdienst (DWD) (DWD, 2016) is used. Records are accessed via the Climate Data Center (CDC) and all stations with records during the studied period are used. The pyranometers installed here are mainly Secondary Standard, the CM11 and CM21 [Kipp&Zonen]. In France, data from Meteo-France, the national meteorological service, is used. Most stations belong to the synoptic network (type 0 stations), but areas with low density of synoptic records are covered by data from automatic stations (type 1). This was primarily the case in the Alps and in the Pyrenees. Finally, in Spain ground records are obtained from the Servicio de Información Agroclimática para el Regadío (SIAR) (SIAR, 2015). SIAR is a Spanish agricultural network maintained by the Ministry of Agriculture, Fishing, Food and Environment. Stations are mainly located in irrigated areas, and solar irradiance is recorded with silicon-based photodiodes, the SP1110 [Skye Instruments]. Only in one station a thermopile pyranometer is used, the Second Class CM5 [Kipp&Zonen].

**Table 1:** Description of the monitoring networks used as reference data. TST stands for True Solar Time, in opposition to UTC, Coordinated Universal Time.

					Type of pyranometer			
Network	Type	N	Interval	Midpoint	Secondary standard	Second class	Photodiode	Not reported
Met Office [UK]	Meteo	121	60 min	:30 UTC	85	9	–	27
LMT [NOR]	Agro	29	60 min	:00 TST	28	1	–	–
SMHI [SWE]	Meteo	12	60 min	:00 TST	12	–	–	–
FMI [FIN]	Meteo	27	60 min	:00 TST	27	–	–	–
DWD [GER]	Meteo	34	60 min	:30 TST	34	–	–	–
Meteo France [FRA]	Meteo	49	60 min	:30 TST/UTC	48	–	–	–
SIAR [SPA]	Agro	33	30 min	:15/45 UTC	–	1	32	–
BSRN	–	7	1 min	–	7	–	–	–
JRC-Ispra [IT]	–	1	1 min	–	1	–	–	–

In addition to these data, seven stations from the Baseline Surface Radiation Network (BSRN) and data from the JRC in Ispra (Italy) have been included. The BSRN stations are well-known for their high quality and all of them use Secondary Standard ventilated pyranometers: CM11, CM21 and CM22/CMP22 [Kipp&Zonen]. The BSRN stations considered are Lerwick (UK), Camborne (UK), Carpentras (France), Palaiseau (France), Payerne (Switzerland), Lindenberg (Germany) and Cener (Spain). The data from the JRC site in Ispra (Italy) is recorded with a CM11 [Kipp&Zonen] and can be freely accessed via JRC (2016).

## 4 Methods

### 4.1 Quality control (QC)

The following quality control tests are applied to the data before being integrated into daily values:

• All night irradiance values are set to 0 (solar elevation angle < 0°).
• Duplicated records are removed.
• The BSRN quality checks (Long and Dutton, 2002) limit tests (“physically possible” and “extremely rarely”) are applied. The “across quantities” procedures cannot be implemented as in most stations only the global irradiance value is available. Due to the high amount of data, all flagged values are automatically set to “Not Available” (NA) and the number of flagged values per station is monitored. Having previously set all night values to 0 reduces the number of flagged values. In general, this number is quite low, being usually under 100 values per station when working with hourly or half-hourly data and 400 cases per station with minute data for the 11 years of the studied period. Only in 5 stations the number of flagged values is above 1000. These 5 cases were manually inspected and corresponded to stations with time shifts and major failures in the recording system with values completely out of the normal intervals.
• Days with no hourly records above 0 are removed. These are periods set to 0 by the data logger when no record is available or just the consequence of electronic problems in the sensor. The polar night is excluded from this check and maintained in the dataset for the validation.

In the satellite products with hourly resolution (SARAH-JRC, operational) the night hourly slots are also set to 0. Besides, negative slots during daytime (solar elevation angle > 0°) are set to “Not Available”. No quality control procedure is applied to neither ERA-Interim nor CLARA-A2 datasets.

### 4.2 Data aggregation and merging

Ground records with minutely and half-hourly resolutions are averaged to hourly values centered at :00 UTC. The aggregation from minutely to hourly is performed following one of the methods described in Roesch et al. (2011). Initially, the 15-min averages are computed from the minutely values. The average is obtained if at least 5 min are available. Then, hourly means are obtained by averaging the four 15-min slots. All four slots have to be available. Half-hourly data is directly averaged into hourly values centered around :00 UTC. Both slots are required to compute the means.

Daily means, either ground or satellite, are obtained by the integration of the hourly values if at least 20 slots are available. As no gap-filling method is applied, missing hourly slots produce a systematic underestimation of daily means. This is a significant effect in the case of the satellite operational products, which has a reduced availability at low solar elevation angles. This effect is analyzed and quantified in Subsection 5.1. Note that calculation of the daily means from the satellite data is done slightly different by the CM SAF which applies the method from Diekmann et al. (1998). The daily means of ERA-Interim are obtained by directly adding the 3-hourly estimates, as the dataset is already quality checked by the ECMWF and it contains no missing values.

Hourly ground irradiance means and hourly satellite slots (SARAH-JRC, CM SAF-Operational) are merged to perform the hourly analysis. The merging is performed by selecting the closest satellite slot to the midpoint of the ground hourly interval. If there is not any satellite slot in the interval ±30 min, the satellite value is considered not available. The time of the satellite hourly slots depends on the image used by the satellite model. Both SARAH-JRC and the operational product use only one of the four Meteosat satellite images available per hour, so the hourly value is actually the instantaneous irradiance estimation. SARAH-JRC computations are made with MFG images at :50 UTC (50 min past the hour) until 2005, and with MSG images at :10 UTC since 2005. The operational product uses MFG images at :55 UTC. These scan times have been calculated for central Europe neglecting the scan time variation between rows of the satellite image.

For the daily analysis, the two daily satellite datasets are included (CLARA-A2 and ERA-Interim). Polar days at high latitude stations in winter (solar elevation angle  <0°) are kept for the validation.

### 4.3 Detection of periods with systematic bias

A second QC test is applied to detect samples that pass the typical QC procedures, such as the BSRN test mentioned in Subsection 4.1, but are still systematically biased and hinder the validation process of the radiation products. These deviations from the real irradiance profile are mostly caused by equipment and operational errors such as a mis-calibration of sensors, electronic failures, shading, time shifts, soiling and presence of snow or frost over the sensor. A novel QC procedure (Urraca et al., 2017) has been designed to identify these types of errors making use of the stability of the satellite models, as these datasets are each calculated with a distinct model and set of inputs for the whole spanned period. Hence, it is possible to characterize the bias of the products at each location, and then flag those periods where the bias is out of the typical values. The specific steps of this QC test are as follows:

1. *Error characterization: definition of the confidence intervals for the daily bias*. The confidence intervals (CIs) are built with the median absolute deviation around the median (Leys et al., 2013):
  (1) CI=median±n×medianabsolutedeviation
  where *n* is a weighting coefficient. The median and the deviation are computed in a monthly basis for each station (monthly aggregated bias of daily means), and subsequently the values obtained are averaged in groups of stations. This attenuates the potential numerical instabilities in the case of too short or completely biased time series. Stations are grouped by country, as they share similar geographical and measuring conditions. Only in the case of the Nordic countries (NOR, SWE, FIN) an additional group is made with high-latitude stations (latitude > 65°) due to the particular conditions of this region.
2. *Identification of biased periods*. The daily bias is compared against the CIs of typical monthly values for all radiation products at each location. A window function flags a group of days if the daily bias of all radiation products is out of the CI in more than 80*%* of the samples compared (days times products) within the period considered. The function is run twice, firstly looking for short periods of high bias (window width = 20 days, n = 2.4), and secondly looking for long periods of medium-constant bias (window width = 90 days and n = 0.4). In both cases, values with a daily absolute bias below 5 W/m^2^ or a relative bias under 5% are neglected.
3. *Quick visual inspection and removal of suspicious records*. Grouping locations for the bias characterization mitigates the effect of outliers, but it also entails that any period or location with a product performance outside the typical limits is flagged. This is the case of locations or seasons known to be problematic for satellite-based models, such as mountainous regions or periods with seasonal snow (Suri and Cebecauer, 2014). Hence, flagged periods cannot be automatically removed and required visual inspection. When a period is flagged in a station two graphs are automatically generated: the time series of the daily bias and the hourly irradiance profile of satellite and ground data superimposed. These two plots enable the detection of false alarms, which are kept for the validation, and the identification of the most probable cause of true errors, which are removed from the dataset.

Radiation datasets used to calculate the bias are SARAH-JRC, CLARA-A2 and ERA-Interim. The operational product is excluded as it does not span the whole validation period and besides, it presents some inter-annual instabilities (see Results 5.3). After visual inspection, flagged periods are eliminated in most cases. False positive periods only appeared in mountainous stations in the Alps and Pyrenees and high-latitude locations with seasonal snow. Table 2 summarizes all the locations where periods with different errors in the pyranometric records where identified.

**Table 2:** Description of the periods removed with the QC method.

	Type of issue	n [days]	Station [days]
Operational error	Snow/Frost	4[112]	NOR-Apelsvoll [28], FIN-Rautavaara [39], FIN-Siikajoki [28] FRA-6094002 [17]
	Soiling	7[2358]	UK-370 [42], UK-1352 [225], FIN-Ilomantsi [95], SPA-GR11 [1799] SPA-AL10 [100], FRA-58218006 [47], FRA-64316003 [50]
	Shading	10	UK-918 [221], UK-56424 [1784], UK-57250 [365], NOR-Kise [83] NOR-Maere [730], SPA-A10 [18], SPA-CS04 [20], ITA-JRC [49] FRA-5183001 [730], FRA-27056003 [123]
	Time shift	1[885]	SPA-BU102 [885]
	Zero periods	4[113]	UK-708 [18], UK-1302 [14], SPA-SE12 [40], SPA-SE13 [41]
	Sensor failure	1[162]	NOR-Sortland [162]
Equipment error	Miscalibration	2[2035]	GER-3028 [577], SPA-J01 [1458]
	Sensor replacement	3[2880]	UK-326 [1418], UK-534 [671], UK-586 [791]

### 4.4 Validation

The daily irradiance means from the four radiation products are validated against the ground datasets. Days with no solar irradiance at all (polar night) are kept in the validation process. The validation of hourly values in the case of SARAH-JRC and the CM SAF operational product cannot be performed, due to the big difference in time between the satellite image and hourly ground records (see Subsection 5.1). The metrics used in the validation process are:

The Mean Bias Deviation (MBD):
  (2) $$MBD=\frac{1}{N}\overset{N}{\underset{i=1}{\sum }}\left({ŷ}_{i}-y_{i}\right)$$
The Mean Absolute Deviation (MAD):
  (3) $$MAD=\frac{1}{N}\overset{N}{\underset{i=1}{\sum }}\left|{ŷ}_{i}-y_{i}\right|$$
The Root Mean Square Deviation (RMSD):
  (4) $$RMSD=\sqrt{\frac{1}{N}\overset{N}{\underset{i=1}{\sum }}{\left({ŷ}_{i}-y_{i}\right)}^{2}}$$

where ${ŷ}_{i}$ and *y*_*i*_ are the predicted and measured values of the variable being modeled respectively, in this case GHI. In addition, the relative versions of these metrics rMBD, rMAD and rRMSD are obtained by dividing the absolute metrics by the mean of observed values.

### 4.5 Software

All computations have been implemented in the freely distributed statistical software R (R Core Team, 2014). The core work of data manipulation and visualization was made with the set of packages from tidyverse (Wickham, 2016). Time series were handled with lubridate (Grolemund and Wickham, 2011) package, while spatial objects were manipulated with sp (Pebesma and Bivand, 2005), raster (Hijmans, 2015) and rgdal (Bivand et al., 2016). Finally, solar position calculations were performed with the functions of the solaR (Perpiñán, 2012) package.

## 5 Results and discussion

### 5.1 Uncertainties in the validation process

The validation of satellite-based estimates with ground data is affected by some uncertainties due to the different origin of ground and satellite values. Ground data is recorded at high sampling rates (sub 1-min) independently of the temporal resolution delivered by the monitoring networks (minutely, half-hourly or hourly). On the other hand, satellite-based estimates are derived from satellite images recorded at lower sampling rates. The highest temporal resolution can be obtained with geostationary satellites such as MSG satellites. This satellites scan the surface every 15 min, though products used in this study based on the geostationary satellite data (SARAH-JRC and CM SAF operational) only use 1 image per hour. This introduces different sources of error in the comparison of hourly values. From the temporal perspective, satellite estimates are point estimates and hence do not account for the atmospheric changes in the interval between two consecutive images. Besides, satellite hourly values have to be compared against the closest hourly ground mean/record because this is the typical resolution released by the monitoring networks (see Table 1), and this introduces a time lag that varies from 0 to 30 min. From the spatial perspective, the satellite data are spatial averages because of the finite size of the pixels. This leads to a sort of temporal averaging in the presence of clouds.

**Fig. 2:**
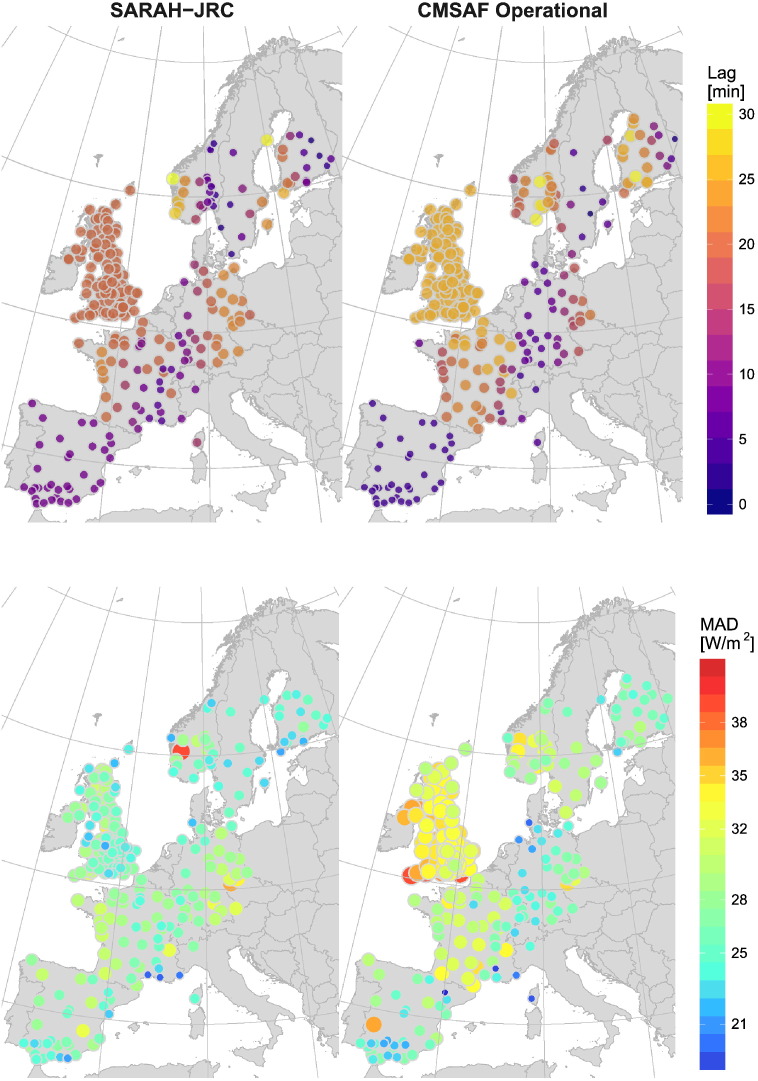
Influence of the time lag between the satellite and ground data in the MAD from 2008 to 2015. Satellite images are scanned at fixed times (SARAH-JRC:10 UTC, CM SAF Operational:55 UTC). Ground hourly irradiance is averaged to different times based on the network. For networks that use solar time, the midpoint of the interval varies for each station and throughout the year (equation of time).

In this study, the time lag between hourly satellite and ground values is the most critical issue. This time lag depends on the midpoint to which the 1-hour intervals are averaged. It varies among the different monitoring networks, but also changes among stations of the same network when values are recorded in solar time. The mean time lag at each station is shown in Fig. 2 along with the hourly MAD for SARAH-JRC and the CM SAF operational product. The figure shows a strong correlation between the time lag and the MAD, which implies that the time lag is the main source of error observed in the MAD spatial distribution. As a result, the initial goal of conducting the validation of hourly satellite estimates had to be discarded. One solution would be to center the hourly values derived from minutely ground records to the minute of the satellite image, but this would reduce the ground dataset to the BSRN and JRC data (8 stations). Another option would be to increase the temporal resolution of satellite estimates to 15-min by processing all available images, reducing the maximum time lag between ground data and satellite values to 7.5 min.

**Fig. 3:**
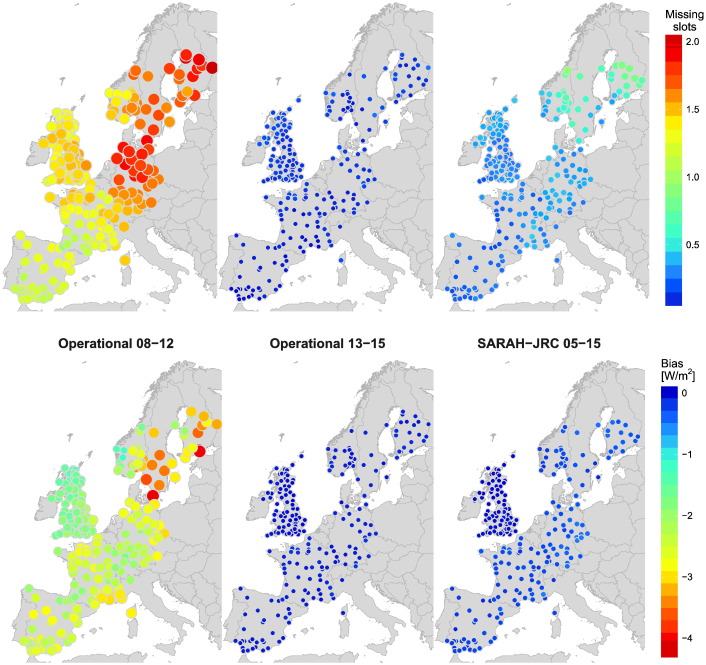
Systematic negative bias in W/m^2^ introduced in the daily satellite means from the integration of hourly slots without using a gap-filling method. The bias is computed in the hourly slots with a solar elevation angle below 15°.

The main source of error in the validation of the daily products used here comes from the integration of hourly values into daily means. A negative bias is systematically introduced in the daily mean when missing hourly slots are present in the radiation products and no gap-filling method is used. These missing values are common in several satellite-based products, as algorithms usually do not cover low solar elevation angles at sunrise and sunset periods. The effect of these missing values is analyzed in Fig. 3, where the number of missing slots is plotted along with the negative bias generated in the daily means. The negative bias is computed by integrating the ground records of the hourly slots with missing satellite values. Only slots whose solar elevation angle is below 15° are used to diminish the influence of random missing slots. The figure proves that the negative bias is negligible for SARAH-JRC (less 1 W/m^2^), as the number of missing values is on average less than 1 in most stations. However, a significant negative bias is observed in the operational product due to the high amount of missing values. This bias evolves with time, as the operational product was updated from 2012 to 2013 by improving the coverage at low solar elevation angles. From 2008 to 2012, the number of missing slots increases with latitude up to an average of 2 missing values in the Nordic countries. These gaps in the time series result in a systematic bias of 2 W/m^2^ on average, peaking 4 W/m^2^ at Nordic countries. It has to be noted that this effect would be even worse if the number of missing slots allowed when computing the daily means hadn’t been limited to 4 (see Subsection 4.2). After 2013, the number of missing values becomes virtually zero, which puts the operational product at the same level of the SARAH-JRC dataset.

**Fig. 4:**
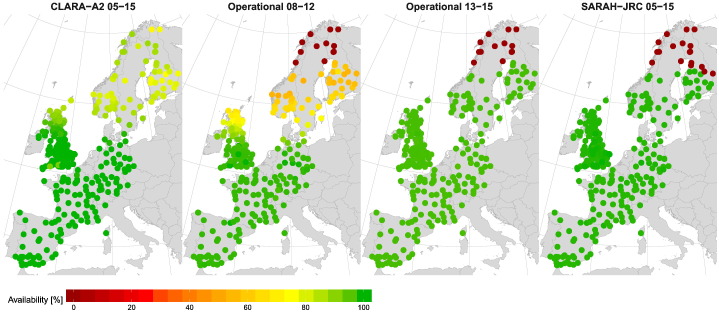
Availability of daily means values of the radiation datasets in the days with valid on-ground records. The maximum number of missing hourly slots tolerated to compute the daily mean was four. ERA-Interim reanalysis has 100% availability at every location.

The availability ratio for the daily means of each satellite-based radiation product is shown in Fig. 4. The availability of ERA-Interim is not included as it has no missing values. The figure shows that SARAH-JRC is the radiation product with the greatest availability ratio, being virtually 100% in all stations within the spatial coverage of Meteosat disk. The CM SAF operational product also has a similar availability after 2013, when the coverage at low solar elevation angles was enhanced. Prior to 2013, the availability gradually decreases above 55° of latitude since the high number of missing slots prevent the computation of daily means. CLARA shows a different availability pattern as it is the only one based on images from polar-orbiting satellites. These satellites have sun-synchronous orbits which results in a different temporal resolution with latitude. They offer around 14 evenly-distributed observations near the poles, while only two observations per day are available near the equator (Karlsson et al., 2016). This is why in latitudes around 65° the temporal resolution available is not high enough to obtain the daily means when the day length is too short. The availability raises again above 65° since the different latitude bands overlap when approaching the polar region. The low availability ratios observed in some cases around 70° are a consequence of the limited coverage of snow-covered surfaces of CLARA-A2.

### 5.2 Validation of daily means

Table 3 summarizes the averaged metrics for each radiation product. The greatest absolute errors correspond to ERA Interim, with MAD and RMSD values almost double of the ones of the three satellite-based products. Moreover, ERA-Interim strongly overestimates solar irradiance, with a positive bias of 8.69 W/m^2^. This overestimation has been reported by other authors (Boilley and Wald, 2015, Bojanowski et al., 2014, Urraca et al., 2016) and it is mainly attributed to the low spatial resolution and an error in the algorithm (Dee et al., 2011). Contrary, the three satellite-based products show a similar MAD between 12 and 14 W/m^2^ and a RMSD between 14 and 16 W/m^2^. The average bias of SARAH and CLARA-A2 is virtually 0, while the operational product shows an average positive bias of 4.47 W/m^2^. This positive bias is specially noteworthy given the systematic negative bias from 2008 to 2012 in the daily means caused by the high amount of missing values in the satellite dataset.

**Table 3:** Validation metrics (mean value ± standard deviation) of the 4 radiation products.

	MBD [W/m^2^]	rMBD [%]	MAD [W/m^2^]	rMAD [%]	RMSD [W/m^2^]	rRMSD [%]
SARAH-JRC	0.68 ± 4.54	0.29 ± 3.84	12.22 ± 2.07	10.09 ± 2.83	17.61 ± 3.18	14.64 ± 4.54
Operational CM SAF	4.51 ± 6.24	4.04 ± 5.57	12.94 ± 2.66	10.79 ± 3.73	18.12 ± 3.64	15.11 ± 5.22
CLARA-A2	−0.83 ± 4.35	−0.49 ± 3.80	13.15 ± 1.87	11.09 ± 2.92	18.64 ± 2.69	15.76 ± 4.39
ERA-Interim	8.69 ± 6.62	7.38 ± 6.15	22.19 ± 3.62	18.59 ± 4.66	33.05 ± 4.88	27.82 ± 7.23

**Fig. 5:**
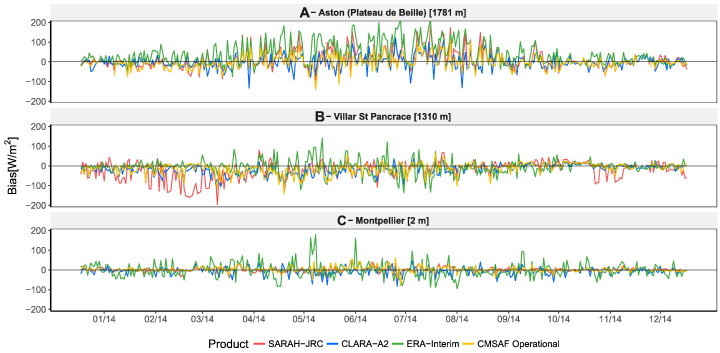
Time evolution of the daily residuals during 2014 at three stations located at different elevations. Station A is located at the top of a mountain in the Pyrenees, station B is located in a valley of the Alps, and station C is located in a flat region close to the shoreline.

**Fig. 6:**
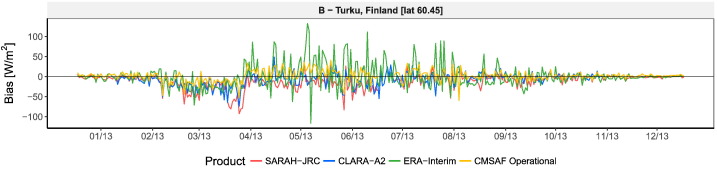
Time evolution of the daily residuals during 2013 in a high-latitude station with a long-snow covered season (January to March).

The five stations located in the Alps and the Pyrenees have been excluded from the computation of the average metrics of Table 3, as the errors are two or three times greater than the ones obtained in most of the other locations. Satellite models fail on mountainous regions because the spatial and temporal resolutions are not high enough to account for the sharp terrain and changing weather conditions. Fig. 5 compares the residual profile during 2014 of two of these stations (A and B) with one station located in a flat region (C). The figure shows how the residuals randomly go up and down in station A, located in the top of a mountain, evidencing the limitations of satellite algorithms on this type of areas. This effect is less evident in station B, located in a valley of the Alps, though a clear underestimation by SARAH-JRC is evidenced during the winter months. This underestimation is also common in regions with seasonal snow, as it is the case of high-latitude countries (see Fig. 6). This is due to the fact that the satellite algorithm only use the visible channel to detect the presence of clouds, and hence cannot differentiate if a bright pixel corresponds to a cloud or to a surface covered by snow.

Due to the high errors in the ERA-Interim product and in the five mountainous stations, these data are hereafter removed from the study to better analyze the trends on the rest of locations and products.

**Fig. 7:**
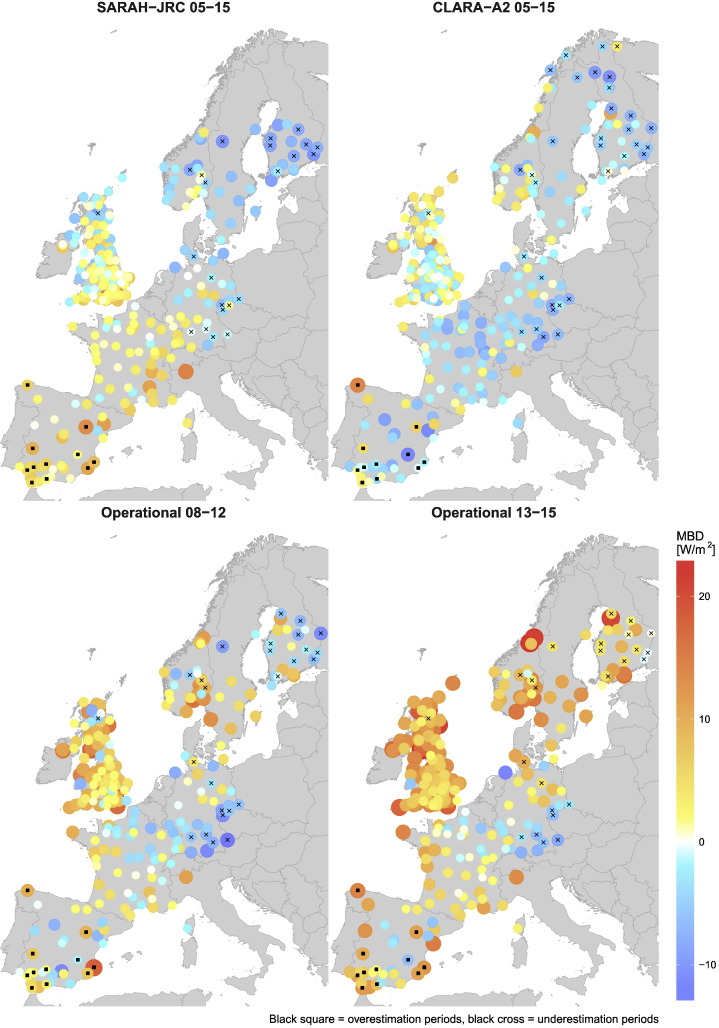
Spatial distribution of the MBD. The operational product is split in two periods due to the increased availability of hourly estimates at low solar elevation angles after 2013.

Fig. 7 shows the spatial distribution of the MBD for the three satellite-based products. The spatial deviations for CLARA-A2 are the lowest, with the product slightly underestimating in most locations over Europe. This agrees with the overall MBD of −0.83 W/m^2^ of Table 3 and with previous validation studies using CLARA-A1 (Karlsson et al., 2012) and CLARA-A1 (Karlsson et al., 2016). However, the validation carried out by Karlsson et al. (2016) over BSRN stations proved that this underestimation has been significantly reduced from CLARA-A1 (MBD = −3.3 W/m^2^) to CLARA-A2 (MBD = −1.6 W/m^2^). The greatest underestimation values are obtained in locations with seasonal snow cover (purple dots), which are abundant at higher latitudes. This contrasts with the overestimation obtained by Riihelä et al. (2015) (MBD = 2.79 W/m^2^) over Sweden and Finland. Despite CLARA-A2 still provides has a limited coverage of snow-covered surfaces, this change is attributed to the higher availability of daily estimates from November to March and to the inclusion of the polar night in the validation metrics. Nevertheless, CLARA-A2 still overestimates at high-latitudes from May to September (see Fig. 12) which was the cause of the positive bias observed in studies using CLARA-A1.

**Fig. 8:**
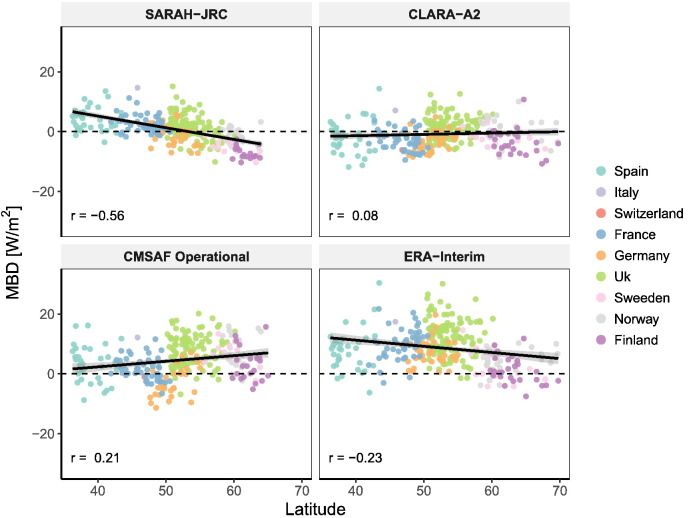
Mean Bias Deviation (MBD) plotted against latitude. The correlation coefficient (r) is shown for each radiation product.

Contrary, the 0.68 W/m^2^ average MBD of SARAH-JRC does not agree with the spatial distribution plotted in Fig. 7. SARAH-JRC varies from underestimation at high latitudes, to an overestimation around 5 W/m^2^ in the South, while unbiased estimations are only obtained in Central Europe (France, Germany and South UK). This pattern is better observed in Fig. 8, where the MBD is plotted against latitude. A negative correlation coefficient of 0.56 is obtained for SARAH estimates, which proves the significant influence of latitude in SARAH-JRC values over Europe. This agrees with previous validations of the SARAH dataset. Riihelä et al. (2015) showed that SARAH underestimates at high-latitudes (Sweden and Finland), with an overall daily MBD of −4.68 W/m^2^, while the CM SAF validation reports at BSRN stations (Müller et al., 2015) proved that SARAH overestimates ant mid- and low-latitudes.

The CM SAF operational product is analyzed in two different periods, 2008–2012 and 2013–2015, to quantify the effect on the MBD of the number of missing values in the satellite dataset. Fig. 7 evidences the systematic negative underestimation during 2008–2012 compared to 2013–2015, due to the high number of missing values in the former period. The increase from 08–12 to 13–15 period in the bias is seen in the majority of locations, though it is more noticeable at high latitudes where the negative bias caused by missing values is higher (see Fig. 3). Once the systematic bias due to missing values is removed, a significant positive bias is observed for the operational product (2013–2015). This overestimation is more prominent in the coast, where the MBD reaches values between 10–20 W/m^2^. On these locations, models have to deal with the presence of land and sea in the same pixel of the satellite image and with particular weather conditions. Besides, it is also noticeable that the operational product is the only one that overestimates at high latitudes, where the presence of snow during winter months typically leads to an underestimation.

**Fig. 9:**
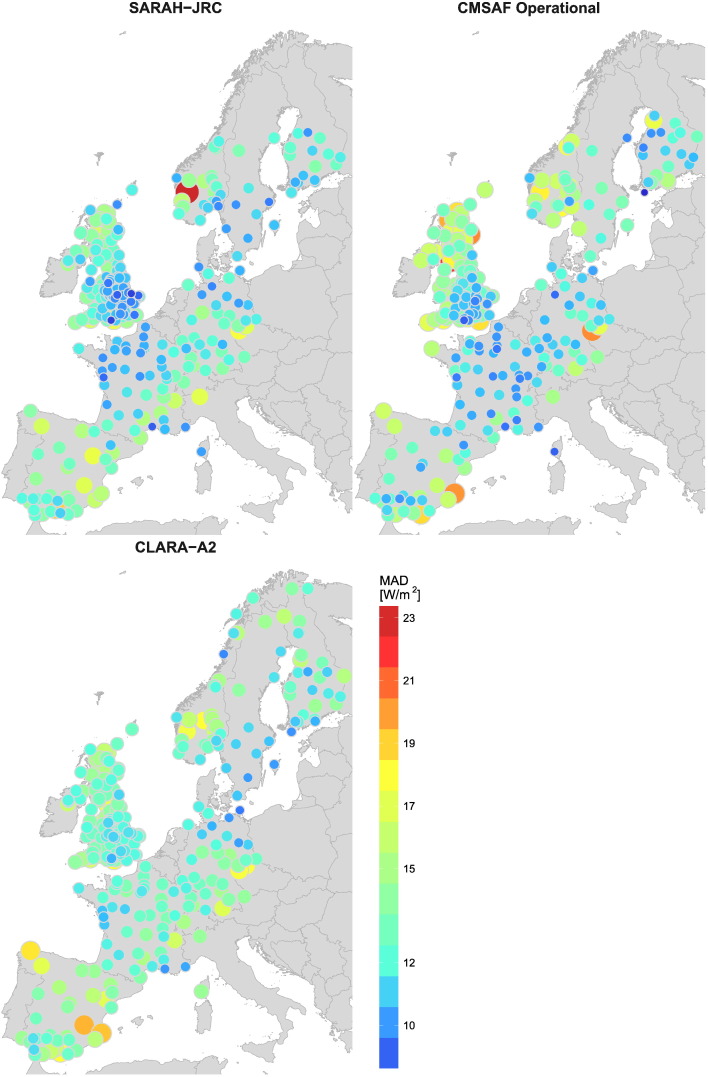
Spatial distribution of the Mean Absolute Deviation (MAD).

Fig. 9 shows the spatial distribution of the MAD. In this case, the study period for the operational product is analyzed at once because the missing slots at low solar elevations do not have a significant influence in the absolute error (figure not shown). Fig. 9 shows how SARAH-JRC and CM SAF operational products, both derived form geostationary satellites, are the ones with the smallest absolute error in most regions. The main difference between both products is found at the coast, where the MAD for the operational product is substantially larger. The spatial distribution of CLARA-A2 is again the most uniform one, but CLARA-A2 cannot match the lower MAD values of SARAH-JRC in Central Europe (France, Germany and South UK). In this predominantly flat region, SARAH-JRC produces the best estimations overall with a MAD between 8 and 11 W/m^2^. The MAD increases in more mountainous regions, such as Northern UK, Norway and the foothills of the Pyrenees and the Alps (MAD = 12–15 W/m^2^). The MAD also reaches the same range in Spain, even though the region apparently presents the most favorable conditions for the estimation of solar irradiance with the greatest amount of clear-sky days. However, the poor reliability of the photodiodes from the Spanish network might be behind the higher and more variable absolute error, as well as the greater absolute value of the irradiance values reached.

### 5.3 Inter-annual stability

**Fig. 10:**
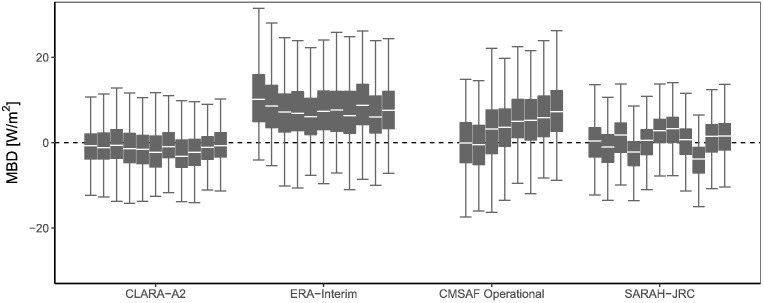
Yearly-aggregated bias of daily values from 2005 to 2015.

Fig. 10 depicts the boxplots with the inter-annual evolution of yearly-aggregated bias for the four radiation products. SARAH-JRC, CLARA-A2 and ERA-Interim show a good temporal stability on the studied period. SARAH-JRC and CLARA-A2 are climatological data records from CM SAF specifically designed for the study of climate trends, and consequently the temporal stability is a critical property of these datasets. The ERA-Interim reanalysis is also designed with the same purpose, and estimations are obtained with a single NWP model and set of inputs. Contrary, an increasing MBD with time is observed in the operational product. Operational products are subjected to different updates, as their main goal is to generate near real-time estimates for climate monitoring applications. In this case, the increasing bias is related to the aforementioned reduction of the number of missing hourly values from 2012 to 2013, though the increasing bias observed is more progressive.

**Fig. 11:**
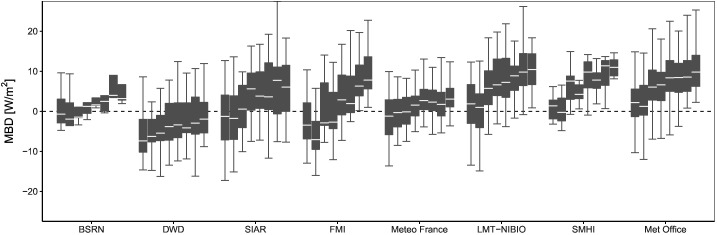
Yearly-aggregated bias of daily values for the CM SAF operational product. Only stations with at least 6 years of data from 2008 to 2015 are included.

The temporal instability of the operational product is analyzed in detail in Fig. 11, where the yearly-aggregated bias is grouped by monitoring networks. The MBD increases in time in all networks, but different patterns are observed. The MBD stabilizes after 2013 in Germany, Spain, France and UK, when the coverage at low solar elevation angles was enhanced. However, in the case of the Nordic countries, the MBD keeps increasing until 2015. Besides, the boxplots again evidence the overestimation in the operational product during 2013–2015, while unbiased estimates are only obtained in France and Germany.

**Fig. 12:**
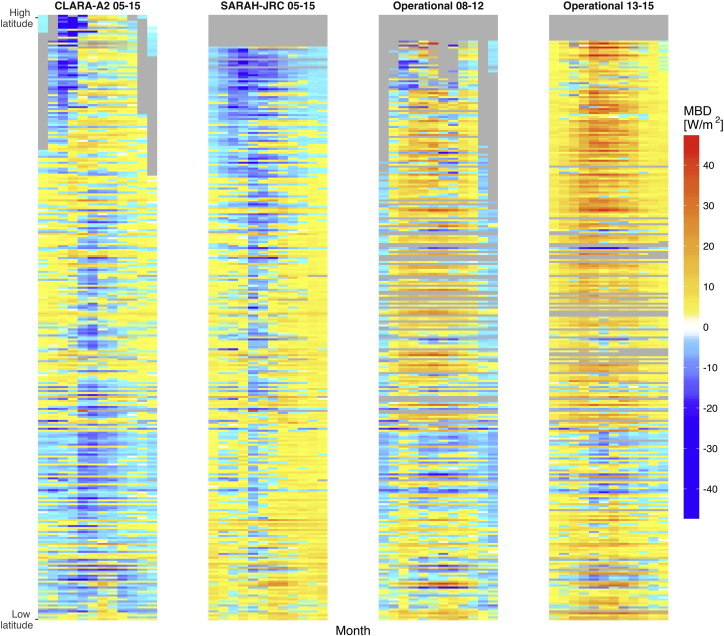
Monthly-aggregated bias of daily values. Stations in the y-axis are sorted by latitude. Only months with 20 valid days are used.

### 5.4 Intra-annual variability

The heatmaps of Fig. 12 depict the intra-annual variation of monthly aggregated MBD for the different stations sorted by latitude. CLARA-A2 and SARAH-JRC present quite similar intra-annual distributions, with underestimation in May-June and overestimation in the remaining months. However, in both products the greatest underestimation is found from January to April at high latitudes, which corresponds to the aforementioned months with seasonal snow. The operational product is again divided into two heatmaps (2008–2012, 2013–2015). These heatmaps reveal that the negative bias caused by missing values mainly affects the period from November to January in Central and South Europe, and the whole year at high-latitudes. These areas, characterized by a high number of low solar elevation hours, turn from blue to yellow after 2013 with the reduction of missing values. However, the intra-annual distribution observed during 2013–2015 in the operational product differs from SARAH-JRC's despite having the same availability ratio. Both products slightly overestimate during winter, but the operational product highly overestimates in summer months. This overestimation is specially significant at high-latitudes, where the MBD reaches 40 W/m^2^ while the average daily irradiance is barely around 100 W/m^2^.

### 5.5 Influence of the type of pyranometer

The influence of the type of the pyranometers on the validation results can be analyzed due to diversity of pyranometers that composed the ground dataset. We mainly focus on the difference between thermopiles and photodiodes, and within thermopiles, the difference between Secondary Standards and Second Class.

The Spanish agricultural network is composed by 32 silicon-based photodiodes and one Second Class thermopile. Photodiodes, based on the photovoltaic effect, are a more economical option to measure solar radiation than the conventional thermopiles, making them suitable for agricultural networks such as the Spanish one used in the present study. Regardless of the absence of Secondary Standard records in Spain, an exploratory comparison can be made between Spanish and French ground records due to their proximity. The main difference in the spatial distributions of the bias between Spain and France is the higher variability observed in Spain despite its more stable climate (higher number of clear sky days). This non-uniformity affects both the bias distribution (see Fig. 7), where the MBD goes from positive to negative in few kilometers, and the MAD distribution (see Fig. 9), which varies from 10 to 17 W/m^2^ under similar terrain conditions. Moreover, random overestimation periods are observed in the residual distributions of most Spanish stations (black dots of Fig. 7). These periods with positive bias in the warm and stable climate of Spain could be attributed to an overestimation during the periods with high aerosol content. However, in this case the periods observed are too long (from 1 to 4 months), and besides they do not coincide with the typical periods of high aerosol content. Therefore, this non-uniformity is more likely to be associated with an inconsistency of photodiodes, as well as to the lower maintenance levels of an agricultural network. Most of these periods have been flagged and eliminated with the QC procedure introduced in Subsection 4.3, making the Spanish network the one with more problematic sensors detected (see Table 2).

**Fig. 13:**
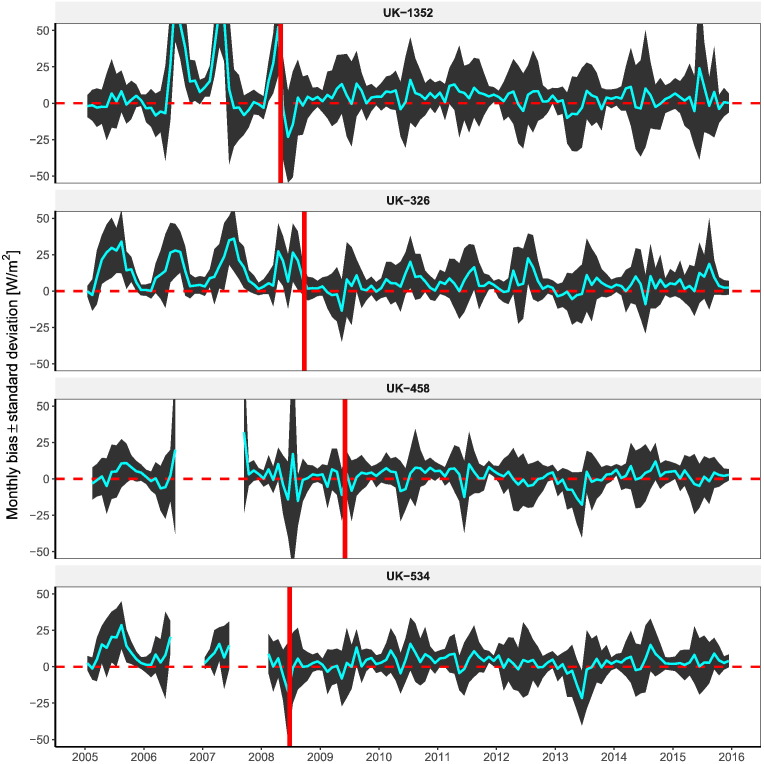
Monthly-aggregated bias plus minus its standard deviation in four stations where Second Class pyranometers were replaced by Secondary Standards. The red vertical line depicts the date of replacement.

The comparison between different qualities of thermopile pyranometers is performed in the British network, as they undertook a major campaign from 2005 to 2010 for the replacement of Second Class pyranometers with Secondary Standards. From the total 121 stations in UK, we have identified 10 stations where the pyranometer was upgraded and with at least one year of data prior and after the replacement date. Fig. 13 depicts the monthly bias evolution of four of these stations, the ones where the change in the quality of ground records was more noticeable after the upgrade. The lower quality of Second Class records is evidenced in different ways such as a high systematic bias (UK-326), a rapidly changing bias (UK-1352) or a greater standard deviation (UK-458). The four periods prior the replacement of Second Class records have been flagged by the QC procedure and subsequently eliminated. It has to be noted that with both types of low-quality sensors, photodiodes and Second Class, the QC procedure was able to identify the worse quality of ground records obtained.

In brief, these differences observed in the validation of satellite-based products between photodides and thermopiles and between Second Class and Secondary Standards prove that the type and quality of the pyranometer is one of the major factors affecting the spatial distribution of errors. This implies that the confidence levels of high-quality satellite-based products such as SARAH-JRC and CLARA-A2 are getting closer to the confidence levels of pyranometers, at least to those pyranometers with greater uncertainties such as photodiodes or Second Class thermopiles.

## 6 Conclusions

In this study we have validated three CM SAF satellite products over an extensive network of 313 pyranometers across Europe. The datasets are two climate datasets, SARAH-JRC and CLARA-A2, and one CM SAF operational product. The ERA-Interim reanalysis has also been included.

First, we have analyzed different sources of uncertainty that can interfere in the validation of satellite-based products. The most influential external factor found has been the quality of the pyranometer used. Stations with Second Class pyranometers and silicon-based photodiodes have obtained not only larger but also more instable validation errors compared to the ones using Secondary Standard sensors. Due to the advances in satellite-based products, the accuracy of low-quality pyranometers is not enough to validate the newest products, and the use of Secondary Standards should be mandatory. Besides, the validation of hourly instantaneous values (SARAH-JRC and CM SAF operational product) have not been possible due to the time lag between the satellite image and the hourly ground records, which is the typical resolution delivered by meteorological agencies. Ground records with minutely resolution, such as the ones of the BSRN, are required to validate hourly or sub-hourly satellite estimates and therefore this study focuses only in the validation of the daily means. In this respect, the presence of missing hourly slots in the integration from hourly to daily has also been quantified, finding that the presence of 1–2 gaps around sunrise and sunset per day results in a underestimation of the daily means up to 4 W/m^2^. This was mainly the case of the operational product due to its limited coverage at low elevation angles during 2008–2012.

Second, the validation of the daily means has evidenced the superior quality of the three satellite products compared to the ERA-Interim reanalysis, which showed a constant positive overestimation and an absolute error almost doubling the one of the satellite datasets. All satellite datasets showed a good performance in Central Europe (France, Germany and South UK), a predominantly flat region where the MAD was within 8–13 W/m^2^ and the bias close to zero. Contrary, the limitations of satellite-based models were evidenced in high latitudes, high mountains, snow, and the coast.

Overall, the validation results over a Europe with a high density of pyranometers have confirmed that SARAH-JRC is the most appropriate product for climate monitoring applications. SARAH-JRC was the most consistent dataset, with the smallest MAD and bias in the majority of locations, whereas the only issue observed was related to the bias distribution, with the dataset underestimating at high latitudes while slightly overestimating in the South. CLARA-A2, showed a good temporal stability as well while keeping a small constant underestimation in the majority of locations. However, the MAD was 1–2 W/m^2^ higher than SARAH-JRC's in most cases, evidencing the higher accuracy of a geostationary dataset compared to a polar-orbiting one. Finally, the operational product was able to reach similar levels of accuracy as SARAH-JRC in most stations in Central Europe, but lacked from the temporal stability of the climate datasets and had more accentuated issues at coastline locations.

## Figures and Tables

**Table A1:** List of ground stations.

Station	Period	Station	Period	Station	Period	Station	Period	Station	Period
FIN - Parainen	05–15	SWE - Vxj	05–15	UK - 1144	05–15	GER - 1358	05–15	FRA - 64549001	05–15
FIN - Kaarina	05–15	SWE - Visby	05–15	UK - 1145	05–15	GER - 1443	05–15	FRA - 66136001	05–15
FIN - Turku	05–15	SWE - Stersund	05–15	UK - 1161	05–15	GER - 1580	05–13	FRA - 67124001	05–15
FIN - Vantaa	05–15	UK - 23	13–15	UK - 1190	05–15	GER - 1684	05–15	FRA - 68205001	05–15
FIN - Helsinki	05–15	UK - 44	05–15	UK - 1198	05–15	GER - 1957	09–15	FRA - 69029001	05–15
FIN - Jokioinen	05–15	UK - 48	05–15	UK - 1205	05–06	GER - 1975	05–15	FRA - 71105001	05–15
FIN - Asikkala	11–15	UK - 54	05–15	UK - 1285	05–15	GER - 2290	06–15	FRA - 73171002	05–15
FIN - Lappeenranta	11–15	UK - 66	05–15	UK - 1302	05–15	GER - 2712	05–15	FRA - 75114001	05–15
FIN - Parikkala	11–15	UK - 67	05–15	UK - 1352	05–15	GER - 3028	05–11	FRA - 80379002	05–15
FIN - Kauhajoki	05–15	UK - 79	05–15	UK - 1378	09–15	GER - 3032	05–15	FRA - 85191003	05–15
FIN - Virrat	05–15	UK - 105	05–15	UK - 1415	05–15	GER - 3098	06–13	FRA - 87085006	05–15
FIN - Jyväskylä	05–15	UK - 113	05–15	UK - 1450	05–15	GER - 3631	05–15	FRA - 95527001	05–15
FIN - Juva	11–15	UK - 132	05–15	UK - 1467	05–15	GER - 3668	09–15	SPA - BA101	05–15
FIN - Seinäjoki	05–15	UK - 161	06–15	UK - 1568	05–13	GER - 3987	05–15	SPA - A10	05–15
FIN - Kuopio	05–15	UK - 163	05–06	UK - 4911	07–15	GER - 4271	05–15	SPA - AB05	05–15
FIN - Rautavaara	05–15	UK - 177	05–15	UK - 17314	05–15	GER - 4336	05–15	SPA - BU102	08–15
FIN - Ilomantsi	05–15	UK - 212	05–15	UK - 17346	05–05	GER - 4466	05–15	SPA - C01	05–15
FIN - Toholampi	12–15	UK - 235	05–15	UK - 18904	05–13	GER - 4642	05–15	SPA - CS04	06–15
FIN - Pyhäjärvi	11–15	UK - 268	05–15	UK - 18905	05–06	GER - 4928	05–15	SPA - HU17	05–15
FIN - Sotkamo	05–15	UK - 315	05–15	UK - 18974	05–15	GER - 5100	05–15	SPA - J01	05–15
FIN - Siikajoki	05–15	UK - 326	05–15	UK - 18993	05–06	GER - 5404	05–15	SPA - M03	05–15
FIN - Oulu	06–15	UK - 370	05–15	UK - 18995	05–07	GER - 5419	05–07	SPA - SE12	05–15
FIN - Rovaniemi	05–15	UK - 384	05–15	UK - 19144	05–06	GER - 5440	05–14	SPA - VA03	05–15
FIN - Sodankylä	05–15	UK - 395	05–15	UK - 19187	06–15	GER - 5705	05–15	SPA - Z18	05–15
FIN - Rovaniemi	05–15	UK - 429	05–06	UK - 19206	05–15	GER - 5779	05–11	SPA - LE01	05–15
FIN - Muonio	05–15	UK - 435	05–06	UK - 19260	05–15	GER - 5792	13–15	SPA - TE01	06–15
FIN - Utsjoki	05–15	UK - 440	05–15	UK - 24102	05–15	GER - 5856	05–15	SPA - HU07	06–15
NOR - Apelsvoll	05–15	UK - 456	05–15	UK - 24125	05–15	GER - 5906	05–11	SPA - SA01	06–15
NOR - Arnes	05–15	UK - 458	05–15	UK - 24942	05–05	FRA - 5183001	05–15	SPA - A03	06–15
NOR - Aasbakk	05–15	UK - 461	05–15	UK - 24948	05–05	FRA - 6088001	05–15	SPA - M02	06–15
NOR - Bo	05–15	UK - 471	05–15	UK - 25046	05–05	FRA - 6094002	05–15	SPA - GR08	06–15
NOR - Etne	05–15	UK - 533	05–13	UK - 25054	05–05	FRA - 8401001	06–15	SPA - J08	06–15
NOR - Favang	05–15	UK - 534	05–15	UK - 25351	05–05	FRA - 9024004	05–05	SPA - SE13	06–15
NOR - Frosta	05–15	UK - 535	05–06	UK - 25727	05–15	FRA - 10228002	05–05	SPA - SE09	06–15
NOR - Fureneset	05–15	UK - 554	05–15	UK - 30437	05–07	FRA - 11069001	05–15	SPA - H10	06–15
NOR - Gausdel	05–15	UK - 556	05–15	UK - 30620	05–15	FRA - 12145001	05–15	SPA - H02	06–15
NOR - Gjerpen	05–15	UK - 583	05–15	UK - 55511	05–12	FRA - 12254001	05–15	SPA - GR11	07–15
NOR - Gran	05–15	UK - 586	05–12	UK - 55827	08–15	FRA - 13054001	05–15	SPA - CO06	06–15
NOR - Gvarv	05–15	UK - 587	05–12	UK - 56214	07–12	FRA - 14066001	05–15	SPA - CA02	06–15
NOR - Hjelmeland	05–15	UK - 595	05–15	UK - 56286	08–14	FRA - 14137001	05–15	SPA - CA06	06–15
NOR - Holt	05–15	UK - 643	05–15	UK - 56424	11–15	FRA - 17300009	07–15	SPA - AL04	06–15
NOR - Kise	05–15	UK - 669	05–15	UK - 56471	09–12	FRA - 18033001	05–15	SPA - AL10	06–15
NOR - Kvithamar	05–15	UK - 676	05–15	UK - 56472	09–12	FRA - 20004002	05–15	SPA - MA01	06–15
NOR - Landvik	05–15	UK - 692	05–15	UK - 56963	13–15	FRA - 21131001	05–15	SPA - MA04	06–15
NOR - Loken	05–15	UK - 708	05–15	UK - 57063	12–15	FRA - 21154001	05–15	SPA - J06	06–15
NOR - Maere	05–15	UK - 719	05–15	UK - 57199	13–15	FRA - 21473001	05–15	BSRN - LER	13–15
NOR - Njos	05–15	UK - 723	07–15	UK - 57247	15–15	FRA - 25056001	05–15	BSRN - CAM	05–15
NOR - Osaker	05–15	UK - 726	05–15	UK - 57250	15–15	FRA - 26198001	05–15	BSRN - LIN	05–15
NOR - Pasvik	05–15	UK - 744	05–15	UK - 57254	15–15	FRA - 27056003	07–15	BSRN - CAR	05–15
NOR - Saerheim	05–15	UK - 765	05–06	UK - 61843	15–15	FRA - 29075001	05–15	BSRN - PAL	06–13
NOR - Sande	05–15	UK - 775	05–15	UK - 61844	15–15	FRA - 33281001	05–15	BSRN - PAY	05–10
NOR - Sortland	05–15	UK - 779	05–15	UK - 61846	15–15	FRA - 34154001	05–15	BSRN - CNR	10–15
NOR - Tjotta	11–15	UK - 811	05–15	UK - 61847	15–15	FRA - 35281001	05–15	JRC - ispra	05–15
NOR - Ullensvang	05–15	UK - 842	05–15	UK - 61915	15–15	FRA - 37179001	05–15		
NOR - Vagones	05–14	UK - 861	05–05	UK - 61937	15–15	FRA - 44020001	05–15		
NOR - Valnesfjord	14–15	UK - 862	05–15	UK - 61938	15–15	FRA - 47091001	05–15		
SWE - Borlnge	05–15	UK - 918	05–15	UK - 61948	15–15	FRA - 49020001	05–15		
SWE - Gteborg	05–15	UK - 1005	05–15	UK - 61949	15–15	FRA - 55386002	08–15		
SWE - Karlstad	05–15	UK - 1007	05–15	UK - 61973	15–15	FRA - 58218006	05–15		
SWE - Kiruna	05–15	UK - 1023	05–15	GER - 183	05–15	FRA - 60175001	05–15		
SWE - Lulea	05–15	UK - 1033	05–15	GER - 656	09–13	FRA - 61169003	05–15		
SWE - Lund	05–15	UK - 1035	07–15	GER - 662	05–15	FRA - 61214002	05–15		
SWE - Norrkping	05–15	UK - 1046	06–15	GER - 691	09–15	FRA - 62516002	07–15		
SWE - Stockholm	05–15	UK - 1083	05–15	GER - 853	05–15	FRA - 63113001	05–15		
SWE - Umea	05–15	UK - 1125	05–08	GER - 1048	05–15	FRA - 64316003	05–15		
